# Semantic TRIZ feasibility in technology development, innovation, and production: A systematic review

**DOI:** 10.1016/j.heliyon.2023.e23775

**Published:** 2023-12-19

**Authors:** Mostafa Ghane, Mei Choo Ang, Denis Cavallucci, Rabiah Abdul Kadir, Kok Weng Ng, Shahryar Sorooshian

**Affiliations:** a Institute of Visual Informatics (IVI), Universiti Kebangsaan Malaysia (UKM), Selangor, Malaysia; b INSA de Strasbourg, 24 Boulevard de la Victoire, 67084 Strasbourg Cedex, France; c Department of Mechanical, Materials and Manufacturing Engineering, University of Nottingham Malaysia, Selangor, Malaysia; d Department of Business Administration, University of Gothenburg, Gothenburg, Sweden

**Keywords:** Semantic TRIZ, Data analytics, Artificial intelligence, Automate innovation

## Abstract

The study unfolds with an acknowledgment of the extensive exploration of TRIZ components, spanning a solid philosophy, quantitative and inductive methods, and practical tools, over the years. While the adoption of Semantic TRIZ (S-TRIZ) in high-tech industries for system development, innovation, and production has increased, the application of AI technologies to specific TRIZ components remains unexplored. This systematic literature review is conducted to delve into the detailed integration of AI with TRIZ, particularly S-TRIZ. The results elucidate the current state of AI applications within TRIZ, identifying focal TRIZ components and areas requiring further study. Additionally, the study highlights the trending AI technologies in this context. This exploration serves as a foundational resource for researchers, developers, and inventors, providing valuable insights into the integration of AI technologies with TRIZ concepts. The study not only paves the way for the development and automation of S-TRIZ but also outlines limitations for future research, guiding the trajectory of advancements in this interdisciplinary field**.**

## 1 Introduction

Semantic TRIZ (S-TRIZ) was pioneered by Verbitsky [1] who semantically combined the meaning of items with traditional TRIZ theory to develop problem and solution patterns. Text mining (TM) and natural language processing (NLP) techniques have the highest relevance for extracting proficient information that is generally applied to classification-related matters [2].

Moreover, technology forecasting (TF) embeds various methods, disciplines, and concepts with combined normative and exploratory methods to determine significant relationships between data [3]. TRIZ, the Russian abbreviation for “Theory of Inventive Problem Solving”, was invented by G. Altshuller's team in the nineteenth century after analysing 40,000 technology patents which revealed a set of patterns for technological evolution [[4], [5], [6], [7]]. TRIZ evolution trends, such as quantitative [8] and exploratory [3] methods have been effectively applied to TF in various fields of technology [5,[9], [10], [11]]. Likewise, in the current era of Industry 4.0, technology development and innovation are undoubtedly essential for organizations across the globe. Nevertheless, it demands a swift response in terms of its digital transformation, as Industry 4.0 alters the major operational systems related to product design, which includes linkages between functional decomposition and morphology (FDM) with TRIZ [12], processes, and services [13].

The use of S-TRIZ within patents has been gaining attention in the automatic extraction of knowledge and information for discovering issues with TRIZ tools [3,14]. In addition, various studies have shown a positive attitude towards automating patent classification by focusing on topic modelling [15] and automating the process for collecting, analyzing, extracting, and interpreting patents using big data techniques [16,17]. The significance of automating and simplifying the analysis of patent documents has gained attention among TRIZ users for several years [18], and this research aims to present a holistic development of what has been achieved so far. This reflects the motivation of the authors to present a comprehensive review of the benefits of TRIZ practitioners and researchers.

This research was conducted to review existing research on S-TRIZ with respect to text mining techniques to help TRIZ developers and researchers maneuver through huge amounts of technical literature [19] so that it can be converted to practical applications of TRIZ tools [7]. Accordingly, a brief report will be presented for each research study, followed by a discussion of the obtained results. Kitchenham, Brereton [20] were adapted to conduct a systematic literature review (SLR) in this study. With reference to the first SLR step, which is “data collection”, the search of the reviewed papers was conducted in the time frame of January 2009 to March 2022, and they were then saved on a local reference manager. In this study, the SLR search was mostly focused on S-TRIZ analysis that used data analysis techniques. Finally, 57 papers were assessed based on the SLR inclusion and exclusion criteria followed by a quality assessment. The contributions of this study are as follows.

• To determine the research on S-TRIZ methodology in terms of data analytics that has been studied between January 2009 and March 2022 (12 years).
• To briefly describe the methods and techniques used for developing and evaluating the S-TRIZ.
• To highlight the limitations of S-TRIZ methods in technology development, innovation, and production.

**Fig. 1:**
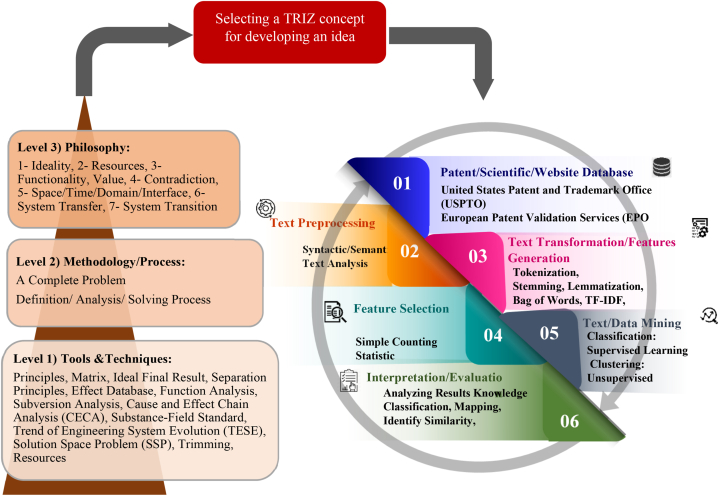
Development of TRIZ theory [7] with S-TRIZ.

TRIZ is classified under systematic innovation and a subset of innovation methods [21]. Recently, Sheu, Chiu [7] presented an updated TRIZ hierarchy that included tools and techniques, methods, and philosophy (with seven pillars), as illustrated on the left side of Fig. 1 Inventors currently apply various TRIZ tools [22] to develop technical systems in which patent analysis is a critical step to meet this objective. Patents provide monopolized knowledge about the technical system. Different methods have been suggested to analyze patent information, such as identifying new technologies, assessing R&D activities, benchmark analysis, ranking patents, and retrieving prior art [23]. Furthermore, patent analysis or mapping based on training materials requires function recognition, keyword searching, document segmentation, abstraction identification, data clustering, result visualization, and data interpretation [24]. Additionally, some common patent analysis techniques exist based on technologies which highlight bibliometric information, the backward and forward relationship among patents citation, statistical approaches, and classification methods [25]. The key features for all types of patent analysis tools that should be considered to satisfy user expectations are listed as follows [26].

• Capability to search and find most related patent in database.
• Reliability to process unstructured texts and transform to structure format.
• Ability to apply different techniques to extract most related information.
• Capability to interact robustly within database during analysis.
• Ability to synchronize with other tools to communicate data.
• Creation of friendly interface including multi-option facility for users.

Fig. 1 indicates the effective text mining procedure in different types of documents to analyze and develop the TRIZ theory.

It commences by accessing a patent database such as USPTO or scientific databases such as the Web of Science, followed by preparing text for syntactic or semantic analysis. In the next step, the text to be tokenized, lemmatized/stemmed, and transferred in the form of features is understood by the machine. After the features have been selected and trained by machine learning (ML) algorithms, the outputs are classified or clustered. The results were interpreted accordingly.

Therefore, bridging the available literature to the existing implementation gap on document analysis, especially related to patents in the context of text mining, would be beneficial for TRIZ practitioners to understand the text mining applications in their product development and innovation processes [7].

However, this would be vital for data scientists to further enhance existing patent analysis based on TRIZ tools [2]. TRIZ is used to offer new ideas and solutions [27]; however, application of computer-aided techniques to develop TRIZ needs to be considered further.

In this study, further steps are organized into four sections. Section 2 explains how research was conducted based on the SLR process. Section 3 provides a brief discussion, highlights the results, and explicitly responds to research questions. Finally, Section 4 presents the research limitations and conclusions.

## 2 Research method

**Fig. 2:**
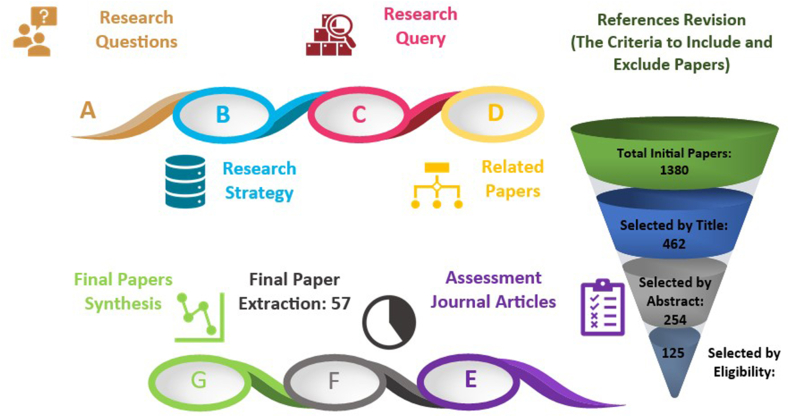
Processes and steps to perform the SLR for S-TRIZ.

Multiple SLRs have been derived for various papers published in different fields [28] and have been validated as a means to objectively diagnose and scrutinize the research issue. Reviewing S-TRIZ systematically provides an opportunity to develop efficient TRIZ tools. As the term implies, a systematic literature review identifies, evaluates, and filters relevant research publications related to pre-determined research questions to provide a detailed overview to researchers and scholars [28]. Fig. 2 illustrates the methodology used to conduct a systematic review of S-TRIZ. The following are the steps in detail.

### 2.1 Define research questions

To conduct this study, the following research questions were designed for further analysis.

RQ1 What studies were conducted from January 2009 to March 2022 in the S-TRIZ?

Our goal for this question is to identify research articles that are most pertinent to advancing TRIZ with AI.

RQ2 What techniques or methods have been used for S-TRIZ?

This question aims to provide a comprehensive understanding of the selected articles by elucidating the specific TRIZ components developed and the application of AI in their development.

RQ3 How can S-TRIZ facilitate the technology development, innovation, and production?

Through this question, we aim to uncover the benefits of integrating specific AI technologies with particular TRIZ components, shedding light on the advancements in engineering systems.

RQ4 What are the state-of-art limitations for S-TRIZ?

By addressing this question, we identify the current limitations in the automation of development, innovation, and production through the integration of AI and TRIZ, taking into consideration the constraints and challenges highlighted in existing studies.

### 2.2 Select research strategy

A conventional research method was undertaken to perform a comprehensive study of existing research resources by predefining the exploratory steps to discover all related and available research by adopting structured research criteria. A systematic literature review was formulated using a structured research procedure to identify the essential materials for every study. Therefore, while considering the necessary SLR protocols, this research takes cohesive action to identify associated papers during a particular period of publication in popular repositories. The selected keywords were associated with TRIZ and patent analysis in terms of text mining in various repositories by placing high emphasis on the research questions. The repositories used as sources were delineated in Table 1.

**Table 1:** Number of articles identified for S-TRIZ.

Publications	Articles after removing duplication	Articles found by title	Articles found by abstract	Articles found by eligibility
IEEE Xplore	32	18	10	4
ScienceDirect	493	140	77	39
Wiley Online Library	45	9	3	2
SpringerLink	149	54	32	17
Web of Science	84	71	38	12
Scopus	176	86	53	26
Taylor and Francis Online	111	40	15	5
SAGE journals	46	10	3	1
Total	1136	428	231	106

### 2.3 Define research query

Searching for relevant articles is a paramount initial stage in conducting SLR. High-quality inputs are required to harvest high-quality outputs. Therefore, the application of appropriate keywords would lead to obtaining the most relevant articles that meet the scope of the research.

**Fig. 3:**
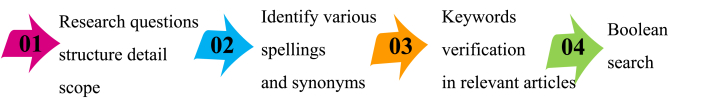
Research process for Choosing relevant articles of S-TRIZ.

The following research keywords were opted for by scrambling the words in permute as “TRIZ” AND (patent OR “NLP” OR “natural language processing” OR “text mining” OR “evolution trends” OR “trend analysis” OR "technology forecast"). The selected query was used to collect information from articles in different publisher repositories. Nevertheless, the keywords were slightly modified based on search engine syntax criteria for various resources. The process of conducting this study is illustrated in Fig. 2. Research options in all resources were left as default to include all types of publications, such as books, journal articles, and others, within the time frame of the studies. It also highlights the duplication removal and filtration steps, as shown in Fig. 2. In addition, Fig. 3 illustrates the detailed procedures applied in searching the articles.

### 2.4 Duplication removal and inclusion and exclusion strategies

Employing the proposed research query in these repositories has resulted in a collection of numerous publications. If we take the example of the Web of Science website loaded under the license of the UKM library, the research query was inputted in the search bar, and the year was chosen according to the research inclusion criteria. The total bibliographic information was extracted in RIS format to export the papers into Endnote reference manager software [29] for ease of management and handling. Accordingly, all these processes were repeated manually in all selected repositories. Endnote has a user-friendly interface that not only displays the article title but also provides easy access to the bibliographic details, groups the references, populates the full text if available, exports them to the CSV file, and demonstrates several helpful capabilities.

Fig. 2 shows that the research steps were conducted holistically, and the details of the initial search and filtration (title, abstract, and content) based on the inclusion and exclusion criteria are depicted. The inclusion metrics that were diagnosed for this research are listed in Table 2.

**Table 2:** Inclusion criteria for Choosing relevant articles of S-TRIZ.

No	Criteria
1.	Papers which were published in January 2009 until March 2022
2.	The article's contents were accessible
3.	The article is in English language
4.	The article spells out the use and application of text mining in TRIZ
5.	The article provides related information to answer the research questions
6.	The articles are available in the mentioned databases
7.	The papers only published as journal article

Exclusion criteria encompass papers falling outside the specified publication range, those lacking relevance to the research questions, written in languages other than English, or published in formats such as books or conference proceedings.

Selection of the relevant papers among the collection of articles was performed in three phases diligently and meticulously. In the initial phase, relevant articles were selected by considering their title relevancy. In the second phase, selected articles from the previous phase were reviewed by reading the abstracts. In the last phase, the remaining articles were carefully read by considering the inclusion and exclusion metrics to identify the most relevant articles.

### 2.5 Defined metrics for quality assessment

To ensure the accuracy of the selected articles and their consistency in meeting the research criteria, the assessment must be conducted effectively. Therefore, the SLR method recommends quality assessment for all publications that were included in the final filtration phase. First, it is essential to define quality metrics for the corresponding research questions. Subsequently, all the evaluated and included publications will be verified whether they match the research questions. The key metrics used to assess this research were as follows.

Metric 1: The chosen publication presents descriptions corresponding to the research questions.
Metric 2: The selected publication provides in-depth information about the techniques used for TRIZ-based data analytics.
Metric 3: The chosen publication explains evaluation techniques for TRIZ-based data analytic.
Metric 4: The chosen publication explains benefits or limitations of TRIZ-based data analytic.

**Fig. 4:**
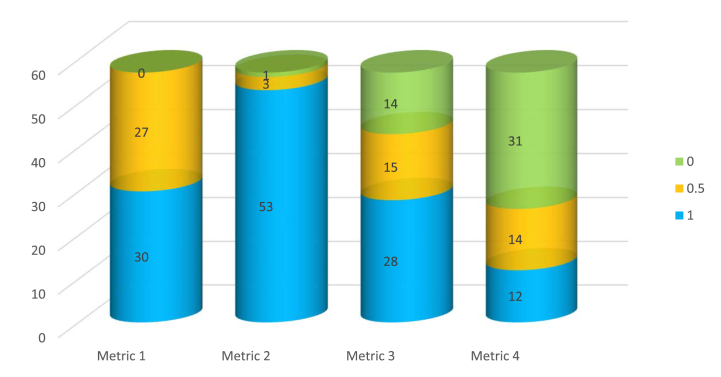
Categorization of chosen articles based on total assessment score.

With this, authors reviewed the chosen publications with reference to the quality assessment metrics. Evaluations of the chosen publications were based on the scoring point of “1” if all the research questions were addressed in the publication, point “0.5” if the research questions were partially clarified and point “0” if there were no explanations provided to address the research questions. Assigned points were then added up to determine the total score for each of the publication. The total score would be a robust value to evaluate and assess the chosen publication corresponding the research questions. Chosen publications further grouped by total score range as shown in Fig. 4 for reliable measure of evaluation.

**Assessment 1:** Out of the 57 papers, 30 provide comprehensive descriptions addressing the research questions, while the remaining publications offer only partial explanations regarding the integration of TRIZ components with AI.

**Assessment 2:** A substantial majority, 53 out of 57 papers, thoroughly elucidate the application of AI techniques in the development of TRIZ, showcasing a comprehensive understanding of the AI methodologies employed.

**Assessment 3:** Focused on the evaluation aspect, 28 papers utilize AI techniques for thorough result assessments, indicating a robust approach. Additionally, 15 papers provide partial evaluations, some of which solely rely on TRIZ techniques.

**Assessment 4:** Regarding benefits or limitations, only 12 papers distinctly discuss either the advantages or constraints of TRIZ-based data analytics. Conversely, the majority, comprising 31 papers, lacks explicit information on this aspect.

### 2.6 Output achievement

The output data in the proposed research were obtained after the assessment process was undertaken on the chosen publications. After fulfilling all SLR requirements, the output achievement is presented in a structured table for better comprehension. The summary is as follows.

**Fig. 5:**
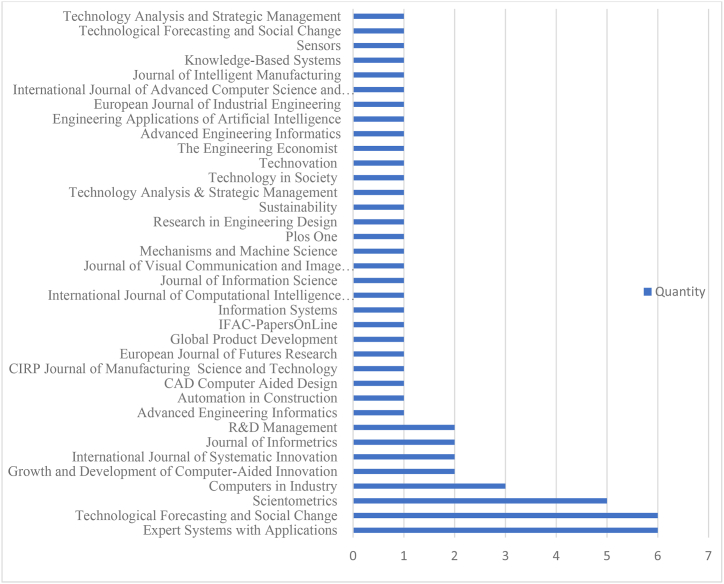
List of journals and number of articles identified for S-TRIZ.
2) Table 4 shows the output from the selection process based on the research criteria, which acts as the fundamental component for retrieving highly relevant articles [20]. It presents the number of articles selected by year and illustrates the patent analysis process based on TRIZ tools using various algorithms and methods.
3) The rest of the paper explains details about information presented in Table 4.

1) Fig. 5 shows the frequency of journals that published TRIZ-based data analytic publications to reflect the importance and role of this research area.

### 2.7 Research argument synthesis

The synthesis was performed to concisely link the research materials. The data extraction procedure is explained extensively in Section 2.4, with a final selection list of 57 publications. Fig. 2 illustrates the research framework according to the research questions, queries, criteria, and outputs. By deep diving into the presented SLR framework, 1136 publications were initially collected in the Endnote dataset. The collected data were then verified individually to ensure that bibliometric information was downloaded properly. Specifically, the initial collection process included several duplications, and some reference sections were missing. Therefore, the collections were cleaned up by removing repeated publications and filling up and correcting the reference sections such as keywords, abstract, publication year, and authors’ name. Subsequently, a group set was created in Endnote with three subset folders named (filtration by title, filtration by abstract and filtration by content) to manually perform the selection stages.

At this stage, 428, 231, and 106 publications were obtained for each stage, respectively. Filtering papers based on the exclusion and inclusion criteria is tricky, overwhelming, and time-consuming. In addition, 106 selected papers were extracted into a CSV file for statistical analysis. CSV files allow numerical and textual data to be saved in a structured tabular format for further analysis. Finally, the articles chosen and cited manually in the next section were used to define the proposed research questions and analyze them in detail.

## 3 Data extraction and analysis

Before delving into the extracted data, we present the acronyms used in S-TRIZ databases, as encountered during the reading of this paper, in Table 3.

**Table 3:** List of acronyms used in S-TRIZ databases.

Acronyms	Description
USPTO	United States Patent and Trademark Office
DII	Derwent Innovations Index
WIPS	Worldwide Intellectual Property Service
WIPO	World Intellectual Property Organization
WFT	Wind Financial Terminal
FPO	Free Patents Online
baiten	A Chinese Database
NBER	National Bureau of Economics Research
JPO	Japan Patent Office
SCIE	Science Citation Index-Expanded
KISTI	Korea Institute of Science and Technology Information
PTR	Public Technological Repositories e.g., technical report, specification, etc.
ITT	Internet Technology Trading
BW	Biological Website
WoS	Web of Science
EI …	EI Compendex
CGP	Chinese Granted Patents

**Table 4:** Assessment based on research questions.

No	The Methods of Selected Publications	TRIZ (Philosophy, Method, Tool)	Database	Pre-processing and Feature Representation	Data Mining Pattern Discovery ML Algorithm	Interpretation/Evaluation
A1.	Applied NLP for feature selection and neural networks algorithm as AI classifier in estimating level of ideality in TRIZ [30].	TRIZ metrics such as degree of ideality and level of invention	NBER	Stop Words Stemming Tokenization (SAO)	• MATLAB Neural Network Toolkit • Support Vector Machine (SVM) • Naïve Bayes	regression coefficient
A2.	TRIZ-based prediction for technical system maturity by analysing patent information with text mining methods [31].	S-curve	patent database	Stop Words Tokenization TF-IDF vector space model	–	S-Curve position of the technology can be determined
A3.	A framework proposed to extract adjectives from patents and then formalize the relevancy with TRIZ evolutionary trends [32].	TRIZ trends eight laws from Mann	(IPC) for specific product	Stop Words Tokenization (adjectives) POS-tagger	–	Visualize trend phase in Radar plot
A4.	Association of rule-based approach on general pattern on TRIZ principles [33].	20 of the 40 Principles	USPTO	Manually select binary set (Training, Testing) from 7 classes, TF-IDF, Co-occurrence	• Support Vector Machine (SVM) • Naïve Bayes (NB) • Decision Tree (C4.5)	Precision Recall F (2) value
A5.	Semantic patent analysis to interpret and classify functions automatically [34].	functional representation TRIZ level of invention	USPTO	part-of-speech (POS) tagging and probabilistic parsing latent semantic analysis Table of citations and target LOI	• Artificial neural network (ANN) • Support Vector Machine (SVM)	Square Error
A6.	Constructed probability of TRIZ evolutionary trends on Radar Plot [35].	40 Inventive Principle	million data-points.	Tokenization (SAO)	–	1 Visualize trend in Radar plot 2 Current/Future IP Value Measurement
A7.	Presented a framework to apply text mining to identify TRIZ evolutionary trend for the management of the R&D [36].	TRIZ evolutionary trends (31)	USPTO	Stop Words Stemming term-pairs, term frequencies	Key Graph diagrams	Evolutionary Potential Radar Plot
A8.	Particular field parameters were extracted from IPC by creating thesauri with NLP techniques [37].	domain technical parameters	USPTO, IPC classes/subclasses	Manually build thesaurus to identify synonymy and hyponymy relationships		Network-based & Excerpt
A9.	Investigated identical properties and functions of product through patent texts [8].	TRIZ evolutionary trends TRIZ trends: space segmentation, surface segmentation, asymmetry, dynamization, controllability, and human involvement. from Mann	US patent IPC: code ‘A45B’	Stemming Tokenization POS: (adjective + noun’ and ‘verb + noun’) Stanford dependency extractor Check similarity between pairs	–	Evolutionary Potential Radar Plot
A10.	“Fact-oriented” patent analysis model was proposed as an automated method [38].	Function-Oriented search	USPTO Free Patent Online	SAO structure, and a fact-oriented ontological approach	–	Case study on function-based Technology Information Retrieval.
A11.	Automated patent classification based of TRIZ level of invention [39].	level of invention	(USPTO) subclass, USPC 360/324,	Stemming used WordNet lexicon part of speech tagging Backward citations measures. frequency of a specific patent against its corresponding corpus term	• Levenberg–Marquardt algorithm • ANN	Mean square F value Pr > F
A12.	“TrendPerceptor” proposed to extract functions and properties within patents by applying NLP [40].	TrendPerceptor TRIZ evolution trends	USPTO)	Stop Words Stemming POS Tokenization (Sentence) computing the similarity of the sentences	–	Radar plot
A13.	“Self-evolutionary model” proposed to recommend substitution for technology automatically [41].	inventive principles (IPs) over the most relevant Engineering Parameter (EP) for the Action link	PTR	Tokenization (SAO) genetic operation tree (GOT) of the target technology	• Genetic Algorithm process • Obtain fitness value	Domain Experts Performance Evaluation
A14.	SAO approach applied for creating properties & functions roadmap [42].	product–function–technology (PFT) maps	WIPS database	Stop Words Tokenization (SAO) (product, technology, material, and technology)	–	R&D Manager
A15.	Automated “Function–Behaviour–Structure (FBS)” model using text mining methods [43].	Components and function analysis Tools, Fields, Artifacts	International patent office's websites	Tokenization (sentence) Stop Words Stemming POS tagging		Network-based & Technicians and Engineers
A16.	An intelligent “Tech Preceptor” framework for strategizing and mapping functions within patent used SAO approach [44].	Function Analysis	patent search engine	Stop Words Tokenization (SAO) Stemming POS features	–	Experts can assess Patent network generator (PNG) SAO network analyser (SNA)
A17.	Prospective and effective patents identified based on TRIZ evolution trends [45].	TRIZ evolution trends	WIPS	NLP process SAO structures	–	Identifying Promising Patents in the Domain by Experts.
A18.	A novel process to convey “Recursive Object Model” to the “Function–Behaviour–State” used for engineering design [46].	help to design methods such as TRIZ	USPTO	ROM (Recursive Object Model), POS (noun (n), verb (v), adjective (a), adverb (ad), determiner (d), preposition (p), and conjunction (C)), PES (Product–Environment System), and FBS (Function–Behaviour–State)	–	Visualizing two-dimensional map for ROM and FBS
A19.	Enhanced word coherency and phrase compilation for “Technology RoadMapping” method to utilized in TRIZ toolkits [47].	TRIZ theory "Problem & Solution" pattern	WoS and EI Compendex	term clumping Tokenization (SAO) M = Materials; T&C = Techniques & Components; P = Products	–	Visualizations of Developmental Trends
A20.	Searching metaphors of functions in patents for conceptual design [48].	Function-based analogy	USPTO	parsing that text Tokenization Stemming vector space model (VSM)	–	Subject-Matter Expert. The Novelty of Ideas Metric
A21.	Identify promise technologies by clustering technical relations between science and patents documents [49].	Fields of Technology	SCIE and USPTO	Stop Words Tokenization vector space model (VSM) Orclus clustering algorithm	–	Cluster Sparsity Coefficients
A22.	Mining promise technologies by developing Technology RoadMap with SAO approach [50].	Technology roadmapping (TRM) with seven layers (material, technology, influencing factor, component, product, goal, and future direction)	WoS	SAO semantic analysis Links components with the most links pointing.	–	1 The Prolific Players for a Component (PP) 2 Research Interest (RI) 3 Novel Technology (NT)
A23.	Exploration of promise technology based on function analysis [51].	Identified occurrence frequencies of each verb in all As and each noun in all Os, and referred to the terms in the function/attribute database scheme of TRIZ	USPTO	SAO structure Semantic functional similarity measurement	–	Semantic Functional Similarity Measurement (Similarity Coefficient)
A24.	A supervised ML technique used for automating patent classification by applying text mining [52].	requirement-oriented taxonomies	USPTO	Tokenization Stop Words Stemming term weighting Information Gain (IG) TF-IDF Vector Space Model (VSM)	• Decision tree (DT) • Naïve Bayes (NB) • Support Vector Machine (SVM)	Accuracy, Precision, Recall and F-measure
A25.	Incorporating functional research and bibliometrics into the technology prediction [53].	S-curves	industry publications, newsletters, websites	Tokenization POS-tagged lemmatization domain–specific terms were weighted with the C-NC value	–	Functional Maps and also big bibliometric data analysis
A26.	Integrated WordNet and Morphology for innovating ideas [54].	Ideation	WordNet lexical information	meronym/homonym for dimension construction and hyponym/hypernym for value construction.	–	–
A27.	Integrated Text mining techniques and rule mining methods to define prospective products [55].	Similar to TRIZ approach	1- existing product database developed by KISTI. 2- (USPTO)	Extracting product information using term frequency or document frequency	–	Aggregation of the Confidence and Weight Value and Correlation Coefficient
A28.	Composition of qualitative and quantitative methods to construct “Technology Roadmapping” based on ST&I information [56].	contradiction matrix	DII	Basic cleaning Fuzzy matching — the stem-based term consolidation Pruning consolidation, calculate the similarity between terms SAO analysis (Label, Implication, Time, Organization)	K-means clustering (Topic clustering)	Expert Knowledge Generate Map
A29.	Measured Identical features such as technical and entity characteristics with semantic patent analysis [57].	TRIZ principals problem/solution patterns	WFT financial database,	Term clumping TF-Infighting	• K-means algorithm • K value of 5 • Co-occurrence	F Measure
A30.	Supervised, semi-supervised and multi-dimension approach used to classify patent automatically by performing Naive Bayes algorithm [58].	functions of products	official patent database	patent text segmentation TF-IDF Information Gain (IG) Mutual Information Vectorization: (feature n: feature weights n)	Naive Bayes	Accuracy
A31.	Integrated F-term technique and patent mining to find emerging invention opportunities [59].	Alternative to TRIZ approach, S-curve	JPO	features are classified by F-term that is technical attributes such as (Purpose, Function, Structure, Material, Methods, Processing and Operation procedure, or Control means)	Cosine Similarity	R^2^
A32.	SAO semantic analysis merged with morphology analysis to discover new technologies [60].	Attribute relationships among products or technologies	DII	term clumping and Principal Component Analysis (PCA)	• Fuzzy matching • Co-occurrence of Subjects and Objects	Magnitude Index , Importance Index (II), Growth trend Index (GTI)
A33.	SAO approach employed to identify patterns between problem and solution to manage R&D development [61].	problem & solution pattern	DII	Stop Words Pruning TF-IDF vector cosine PrincipalComponent Analysis (PCA) SAO semantic analysis	• Fuzzy set matching • Co-occurrence Matrix	Organisation Correlation Map (Network)
A34.	SAO extracted syntactically to identify principle components of system [62].	‘S’ shaped curve Technological components are a series of technology processes, operation methods, functions, and material treatments	DII	SAO cleaning and consolidation Cooccurrence algorithm frequency statistics of patents containing specific requirements, Latent Dirichlet Allocation (LDA)	• Fuzzy Matching-based Consolidation • Co-occurrence	1- Frequency Statistics 2- Correlation Map 3-Relevance Map
A35.	Semantic investigation of the relationship between new feature attributes gleaned from various product [63].	“Reasonable” function attributes combine with TRIZ	USPTO	Stop Words Verbs represented functions. POS tagging LDA algorithm	–	Semantic Similarities Between Designs Functions
A36.	A supervised approach to identify technical patent information automatically [64].	Technical feature ontology	WIPO	binary-syntactic relationships List of lemmas pool of Syntactic Dependency Pattern Unsupervised coarse classification	Coarse Classification	Precision Recall
A37.	A semi-supervised approach for analysing layered technical knowledge in scientific publications [65].	problem solving Extend to tech mining	WoS database	Tokenization (SAO) POS tagging	Cohen's Kappa Coefficient (Statistic)	Precision Recall F-score
A38.	Extended latent semantic analysis model to solve the severe data sparsity in short texts [66]	40 Inventive Principles, 76 Inventive Standards, 11 Separation Methods	Short Texts in TRIZ Knowledge Sources	Tokenization, Stemming, Lemmatization, TF-IDF, Term-item Semantic Matrix, WordNet, Semantic Similarity	Latent Semantic Extraction	Precision, Recall, F1-value
A39.	An Enhanced SAO networks approach based on trend of technical development for graphene [67].	problem and solution Semantic TRIZ	DII	Stop Words SAO Network Construction TF-IDF	Fuzzy matching	SAO network based on Graphene Technology Patents.
A40.	Innovative TRIZ problem-solving theory by using “experience capitalization” [68].	Alternative classical TRIZ problem solving by solving the specific problem directly	USPTO	Tokenization Stemming Stop Word Latent Semantic Analysis (LSA) TF-IDF Problem features Solution features	Co-occurrence Cosine Similarity	ISO 9241-11 Standard (Efficiency, Effectiveness)
A41.	An artificial intelligence based data-driven approach for design ideation [69].	alternative ideation tool	web crawling from Wikipedia	Tokenization Stop Word in Stanford CoreNLP Generative adversarial networks model	–	Consensual Assessment Technique (CAT) was utilized for measurement of novelty, quality, and variety.
A42.	Demand identification model of potential technology based on SAO structure semantic analysis: The case of new energy and energy saving fields [70].	refers to the function of the component	ITT platform in China.	Dependency parsing of the XML structure. segmentation Stop Words POS tagging SAO structure similarity matrix Technical map and demand layout	Segmentation tools (Stanford Parser, Jieba, LTP, Baidu, ICTCLAS)	Accuracy Recall F-value
A43.	Detect technical opportunities (“elements/fields and purposes/effects”) by applying topic modelling through SAO approach [71].	39 engineering parameters of TRIZ	USPTO	Eliminating punctuation, transforming lowercase letters to uppercase lemmatization Stop Words Tokenization (SAO) POS Topic modelling: Latent Dirichlet Allocation (LDA)	Correlated Topic Model	Patent mapping results to select promising topics on Elements/fields & purposes/effects
A44.	A Novel Biomimetic Design Method Based on Biology Texts Under Network [72].	TRIZ 40 principle Contradiction Matrix	BW	Scrawling Stop Words Stemming vector space (Word2Vec) POS tagging (bi-LSTM layer and a CRF layer) TF-IDF	Neural Network Model	Case study on Mapping Tree Model of Engineering Object
A45.	Semantic patent analysis with the integration of “morphological analysis” (MA) and “unified structured inventive thinking” (USIT) [73].	Unified structured inventive thinking (USIT) (simplified from TRIZ) Technology Domain	WIPO Patent Scope	Word Segmentation Stop Words TF-IDF Stemming Object-attribute-function (OAF) Woed2vector F-term	–	Morphology Matrix with Experts' knowledge
A46.	Developed SAO to vector concept based on Document to vector embedding algorithm [19].	function analysis compares the clustering results using the embedding vector with the IPC codes (technology field)	USPTO	SAO structures POS tagging Doc2vec SAO2Vec	Spectral clustering algorithm, which is a graph-based technique	Accuracy
A47.	Iterative semi-supervised algorithm to classify TRIZ based information automatically [74].	Function Analysis	baiten	1666 patents were firstly read and labelled by experts according to the functional basis. TF-IDF	• Used EM (expectation and maximization) algorithm • Naive Bayes	Functions classification Accuracy
A48.	Deep learning technique used to analysis patent's phrase semantically to detect technology opportunity [22].	general answers to research problems	DII	(features, behaviours, or attributes) LSTM network TF-IDF word2vector	K-Means package	ODI-based (Outcome-Driven Innovation) Calculation
A49.	Apply patent mining to integrate TRIZ scientific effects to the patents to create a conceptual design framework [75]	TRIZ Scientific Effects (TRIZSE)	USPTO	Tokenization Stemming, Removal of Characters, Lowercasing Capitals, Stop Words,Doc2Vec, SAO Semantic Analysis	–	Conceptual Design Software Interface
A50.	“Sensors” and “applications” classification and Bipartite Network of Interest (BNOI) construction [76]	Inspired problem & solution	WoS	Tokenization Stemming, N-grams, Word2Vec, BERT, etc.	• Naïve Bayes (NB) • Support Vector Machine (SVM) • Logistic Regression (LR)	F-measure
A51.	A new computational method for capturing effect knowledge to facilitate product innovation [77]	TRIZ Effect's Functionality	WIPO	Delete Category Information, Remove References, Solely defined for Stanford dependency parser (SDP),part-of-speech (POS),	• Recursive Neural Network (RNN) • Long Short-Term Memory (LSTM)	Precision, Recall and F-measure, Experts
A52.	Emerging technology forecasting and evaluating processes [78]	Evolution Law	Chinese Granted Patents	Tokenization, Word2Vec, TF-IDF, Semantic Similarity Analysis	Construction of Supply and Demand (S&D) Matching Model	S&D matching diagram
A53.	Manhattan LSTM is integrated into inventive design solutions [79]	Inventive Design Method (IDM)	USPTO	Bag-of-words, TF-IDF, Patent extractor	Bidirectional LSTM Neural Network	Expert Evaluation, Computation Consumption
A54.	Technology assessment with combination of tech mining and semantic TRIZ [80]	TRIZ Functional Bibliometric Analysis	WoS, PatStat	Syntactic-Semantic TRIZ-Based Tool (Gold Fire)	Tech Mining	Expert Judgment
A55.	A holistic method for the development of complex products [81]	Evolution Law	Granted Patent	NLPIR Parser	Artificial Neural Networks (ANN)	Kendall's coefficient, Accuracy, Expert Judgment
A56.	Apply NLP and AI to retrieve information for Inverse Problem Graph (IPG) [82]	Inventive Design Method (IDM)	Springer, Science Direct, IEEE Xplore	Tokenization, Stop words, Doc2Vec, Cosine Similarity	–	F1-score
A57.	Apply NLP to extract the key components of IDM. automatically [83]	Inventive Design Method (IDM)	USPTO	Tokenization, bi- and tri - grams, Stop Words, Lemmatization, Doc2vec, Latent Dirichlet allocation (LDA)	Affinity Propagation	Precision, Recall, and F-measure

The 57 journal articles cited in Table 4 were chosen to define the proposed research questions and analyze them in detail. The main information extracted from the selected publications was summarized to illustrate the bridge between the TRIZ tools and TM techniques. The retrieved information offers varied scope for both TRIZ tools and TM.

### 3.1 Synthesis of practical applications

In the pursuit of synthesizing the extensive body of knowledge encapsulated in 57 articles at the intersection of TRIZ (Theory of Inventive Problem Solving) and AI (artificial intelligence), a thematic practical analysis has been conducted to distill key insights and trends. This analysis, as presented in Table 5 of the associated paper, unveils five overarching themes that underscore the integration of TRIZ and AI, each encapsulating a unique facet of the amalgamation. From the development of automated technology intelligence systems and TRIZ trend identification to patent classification, knowledge extraction, and ontological approaches, these themes showcase the practical applications and outcomes arising from the synergy between TRIZ principles and advanced AI methodologies. This comprehensive thematic analysis not only serves as a compass for navigating the multifaceted landscape of TRIZ and AI integration but also provides a nuanced understanding of the practical implications witnessed across diverse realms of technology analysis and innovation**.**

**Table 5:** Integration of TRIZ and AI for practical results.

No	Theme	Elaboration	Related Articles
1.	Automated Technology Intelligence and Analysis	Focus on AI-driven systems for technology analysis, leveraging NLP and ML from patent documents.	[41,64,71,79,82]
2.	TRIZ Trend Identification and Mapping	Integration of TRIZ with AI for automated trend identification and mapping, offering an efficient alternative to manual intervention.	[38,46,54,69]
3.	Patent Classification and Knowledge Extraction	Application of AI for patent classification, knowledge extraction, and enhancing creativity in engineering design using NLP and ML.	[35,55,58,66,73,76]
4.	Technology Forecasting and Maturity Assessment	Discussion on methodologies and tools for technology forecasting, integrating TRIZ principles with AI for assessing technology maturity.	[19,32,45,60,77]
5.	Ontological Approaches and System Modelling	Exploration of ontological approaches, fact-oriented modelling, and system analysis integrating TRIZ principles with AI techniques.	[39,48,57,65]

### 3.2 TRIZ tools

TRIZ masters have defined a set of versatile tools for decades of systematic innovation development [4,5,84]. Most researchers believe that using TRIZ tools manually is time consuming, tedious and in many cases unintelligible because they are faced with a large amount of textual information often in the form of patents [22,39,85]. Consequently, to increase the precision and facilitate the utilization of TRIZ tools, integration with artificial intelligence (AI) techniques is essential, as shown in Table 4.

**Fig. 6:**
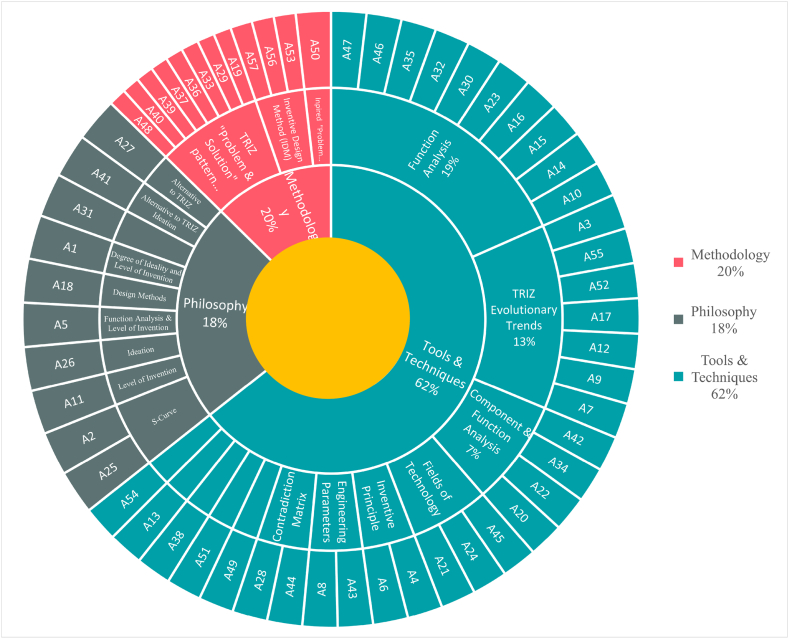
Triz philosophy, methodology, and tools classification.

The application of AI in TRIZ is developed at three levels (Philosophy, Methodology and Tools), as depicted in the TRIZ pyramid in Fig. 1. The classification of selected articles based on TRIZ levels showed that 62% of publications discussed TRIZ tools. The most popular tools, as illustrated in Fig. 6, include function analysis, evolutionary trends, and component analysis, with 19%, 13%, and 7% of the total research, respectively.

The fundamental philosophies that form TRIZ concepts include the seven main pillars of TRIZ; detailed explanations can be found in Ref. [7]. The fundamental pillars (ideality, resources, function value, contradiction, space-time-domain interface, system transfer, and system transition) facilitate ideation [7].

Recently, AI has accelerated the ideation of innovative design; in our study, 18% of the articles fall under this category. It is worth noting that TRIZ tools and methodologies are based on fundamental thinking philosophies [7].

TRIZ methodology seeking identical problem-solution pairs to solve a specific problem that may be applied in different technical fields [86]. Cavallucci and Strasbourg [87] developed an inventive design method (IDM) to expand the TRIZ body of knowledge. IDM aimed to identify initial problems; partial solutions link to possible “cause & effects” are shown in the representation of a problem graph [88].

As a pioneer in the utilization of AI in TRIZ methodology, Cavallucci, Rousselot [89] proposed a framework to extract patent knowledge and combine it with expert knowledge to construct inventive design ontology. Recently, ML techniques have been studied to assist in the development of IDM for A53 and A56. In A57, Berdyugina and Cavallucci [83] took one step ahead in automating the extraction of the key components of the IDM by applying NLP techniques and affinity propagation as ML algorithms.

Recently, AI has accelerated the ideation for innovative design whereby in our research 18% of the articles fall under this category. It is worth noting that TRIZ tools or methodology are based on these fundamental thinking philosophies [7].

The TRIZ methodology seeks identical problem-solution pairs to solve a specific problem that may be applied in different technical fields [86]. Cavallucci and Strasbourg [87] developed an inventive design method (IDM) to expand the TRIZ body of knowledge. IDM aimed to identify initial problems; partial solutions link to possible “cause & effects” are shown in the representation of a problem graph [88]. As a pioneer in the utilization of AI in TRIZ methodology, Cavallucci, Rousselot [89] proposed a framework to extract patent knowledge and combine it with expert knowledge to construct an inventive design ontology. Recently, ML techniques have been studied to assist in the development of IDM for A53 and A56. In A57, Berdyugina and Cavallucci [83] took one step ahead in automating the extraction of the key components of the IDM by applying NLP techniques and affinity propagation as ML algorithms.

TRIZ tools were developed to determine technical conflicts, innovation principles, and function analysis, and to recognize the evolution of systems. For instance, to design Smart Neck Helmets, 39 general engineering parameters were used to identify the design conflict, after determining the contradictions and finally selecting the proper innovation method in the innovation principles [90]. The computerization of TRIZ tools has been an attractive area of research, and this is portrayed in Fig. 6, as 62% of the articles have been mentioned in this category.

### 3.3 Data sources

**Fig. 7:**
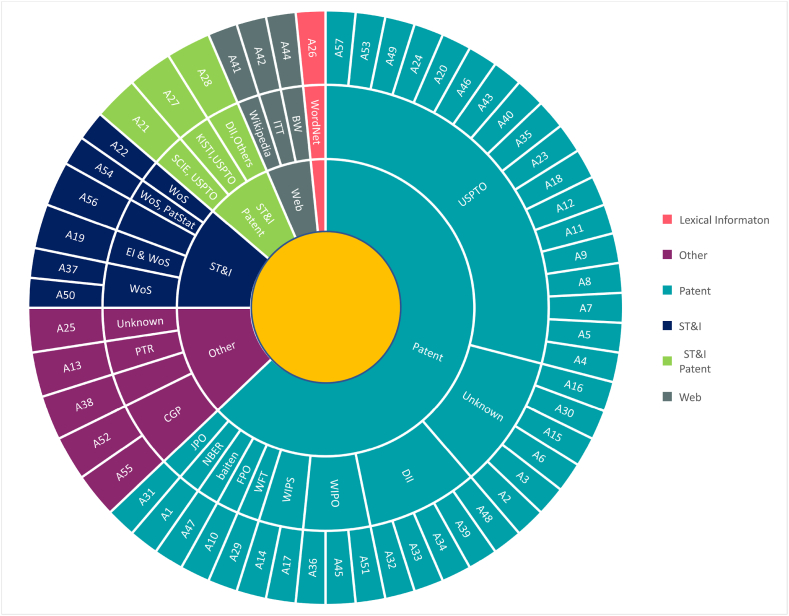
Databases utilized in S-TRIZ.

Data sources are critical in the use of TRIZ. As mentioned in the introduction, TRIZ itself was first formed by analyzing patent files. Nevertheless, applying TRIZ tools for any reason depends on the technical data. Traditionally, TRIZ practitioners have various difficulties searching for pertinent patents and extracting technical information for further analysis. This is why researchers have taken action to facilitate the process of patent analysis by employing the latest computer science technologies and making it as automatic as possible. In our investigation, we focused on two elements (document and database types). In terms of the document type, we identified five different document types in data analytics for S-TRIZ, as illustrated in Fig. 7: (1) patent; (2) science, technology, and innovation (ST&I); (3) web-based; (4) lexical information; and (5) other types of documents, such as newsletters, industry publications, international patent office websites, and manufactures portfolios. Patent documents are the most common type of data used in S-TRIZ activities. Patent databases vary and almost each country has a specific local patent database [91]. USPTO and DII are two popular databases utilized in most S-TRIZ articles. Table 3 presents the list of acronyms used in Fig. 7, which are related to the databases.

Patent documents include structured and unstructured textual data, and S-TRIZ was applied to automate the classification process based on the International Patent Classification (IPC) and Cooperative Patent Classification (CPC) significantly [92]. Experts meticulously consider identifying TRIZ metrics within a patent, such as degree of ideality, level of invention (LoI), S-curve stages, trend of evolution, 40 inventive principles [93], and contradiction matrix [94] have been considered by experts meticulously.

ST&I databases are progressively being considered in seeking newly emerging science & technology (NEST) innovation aspects for decision makers in R&D projects [47,95]. Therefore, quantitative approaches in line with text-mining techniques converge to retrieve functional information from ST&I documents using a tech-mining approach [47,96,97]. However, the semantic TRIZ methodology in terms of technology forecasting and system's evolutionary trend has been a part of the research within ST&I information [47,98].

Web-based resources provide rich information about design systems, demand, and other technical knowledge, such as Wikipedia [69], Internet technology trading platforms [70] and biological data [72]. This information can be retrieved using crawlers and scrapers for further analysis. Lexical information such as nouns, pronouns, verbs, and adjectives is gathered based on their definition in a popular dataset called WordNet [99]. WordNet has been used to generate ideas through morphological analyses [54].

Despite the aforementioned databases, the varied technical information resources provide a system's design details in the form of portfolios or industry publications. Portfolios are a collection of information about a system's design, which provide clear concepts for innovation. Industrial publications provide a broad spectrum of tech-centric outlooks in the form of magazines, websites, newspapers, etc.

### 3.4 Pre-processing and feature representation

NLP and machine learning (ML) are two dominant subcategories of artificial intelligence (AI) utilized in S-TRIZ widely [69,100]. NLP is a confluence of AI and linguistics that intelligently facilitates text analytics [34]. ML is a set of algorithms that enables the statistical solving and analysis of NLP problems by converting unstructured text into a structured format [34]. Therefore, the application of ML and NLP in the context of TRIZ is to automate the processes of understanding language related to the components of engineering systems in textual documents for problem solving and product innovation.

Pre-processing of text documents as an initial step in NLP commonly involves converting text into a format that is measurable, quantifiable, and computable [45]. The most typical preprocessing techniques applied in S-TRIZ are segmentation or tokenization, removal of stop words, stemming, and lemmatization [30,40,71]. The software that facilitates the abovementioned techniques is Python NLTK, VantagePoint, VOSviewer, and WordNet for mapping and so forth. Utilizing a proper preprocessing technique is highly dependent on how noisy a document is and what the expected outcome is. Therefore, the use of these software differs for different projects. In most studies, preprocessing and morphological analysis are used interchangeably [54,97].

**Fig. 8:**
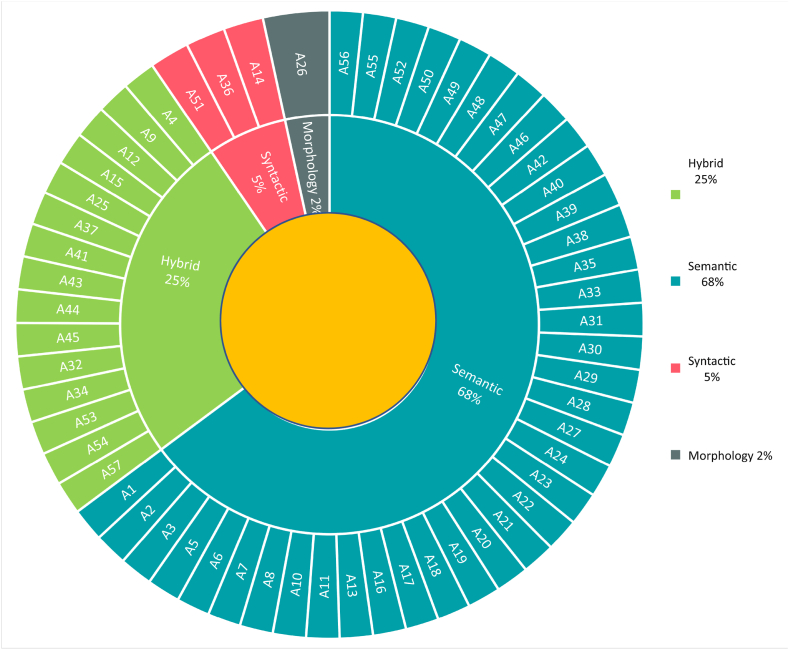
Primary NLP rules in S-TRIZ.

To analyze text documents, natural language processing techniques have been proficiently applied to extract technical features. Two linguistic techniques, syntactic (syntax) and semantic analysis, have assisted machine translation and information retrieval [97]. Syntactic analysis refers to the grammatical linguistic rules that lead to the well-known subject, action, and object (SAO) structure in S-TRIZ [65]. Part-of-speech (POS) tagging techniques are primarily used for syntactic analysis. Semantic analysis contributes to the logical meaning of words and sentences for computers in a manner that a human understands. Fig. 8 shows that 68% of the chosen articles in Table 4 attempted to process semantic analysis, as opposed to syntactic analysis. It also shows that 25% of the chosen articles applied both syntactic and semantic analyses in their studies.

Feature selection in S-TRIZ has applications similar to those in other fields such as text mining and image processing. This process ameliorates the feature or term subset selection with the highest discriminative rate and lowest dimensionality [101].

**Fig. 9:**
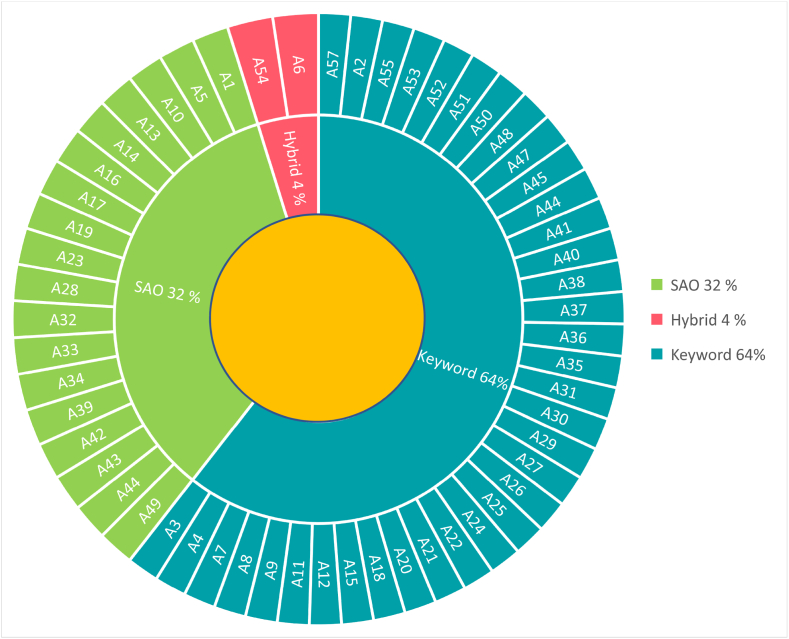
Type of feature selection in S-TRIZ.

Feature selection methods are primarily used in text classification to improve accuracy, reduce dimensionality, and alleviate irrelevant data [101]. The diversity and importance of feature selection methods, including strategies, approaches, types of targets, and labelled data dependency, have been reviewed in detail by Ref. [101]. However, there are two common types of feature selection, namely, SAO structure and keyword-based, which have been identified within the selected articles. Fig. 9 shows that 64% of them were keyword-based, and 32% of articles attempted to extract SAO in their studies. Mann [35] proposed a keyword-based analysis to assess the current value of patents by identifying strength factors and SAO analysis in estimating future value by investigating function words.

There are also several different techniques for selecting either keywords or SAO, depending on how informative they are. Word embedding is a way to represent text as a numerical vector for unique word selection [19,101]. The vector space model (VSM) is a Word2Vec model that represents a document as an array of numbers (vectors), which is based on a similarity score between vectors and calculated by a cosine similarity score [48,49].

The most elementary technique for text vectorization is bag of words (BoW). This model creates vocabulary from all dissimilar words in the corpus and then marks their occurrence as a table of 0 and1 for each sentence [102]. However, BoW has limitations such as the size of vocabulary, complexity in the computation of sparse representations, and neglecting the meaning of words [103]. Therefore, Word2Vec demonstrates two models: (1) continuous bag of words (CBOW) by predicting the current target word according to the source context words, and (2) skip-gram as an unsupervised model predicting the most related words for a current word [19,73].

Another common text vectorization technique is term frequency-inverse document frequency (TF-IDF), which statistically measures the relevance of a word to a document [52]. In fact, two different metrics are multiplied to obtain the weight of words in a document [31,67]. The first metric is “term frequency,” which implies the importance of a word within a document by counting the frequency of a word occurrence. The other metric is “inverse document frequency,” which implies that the measure of a word is common or uncommon within a document. It is a logarithmic formula which results in a rate between 0 and 1 [31]. The results near 0 indicate a common word; conversely, if the results are close to 1, it implies an uncommon word [57,58,73].

VSM models possess limitations from the aspect of inspection of documents owing to the dimensionality and sparsity whereby numerous features are reflected with zero values [97]. One measure to address these limitations is the application of principal component analysis (PCA), which allows dimensionality reduction. This is achieved by converting high dimensionality of vectors to a minimum value in case of sparsity [60,61].

Feature selection based on the SAO structure is a type of document representation in which features are selected syntactically, followed by the indication of subject (noun), action (verb), and object (noun) [44]. Indeed, the SAO structure refers to the TRIZ technical concept and can provide more information than keyword analysis [42,44]. Keyword-based analysis typically focuses on system components that cause the verbs of phrases that imply the function of the system that has been neglected, and consequently, the relationships between components remain intact [62].

In contrast, SAO structure enables scholars to seek core technological aspects creatively [62]. The application of the SAO structure in the selected articles is presented in Table 6. It shows how a technical phrase is interpreted in a text document, what type of information can be extracted, and the type of knowledge obtained after analysis. Generally, the subject and object in a sentence refer to the components or subcomponents of a system, and the action refers to the function and relationship between components.

**Table 6:** SAO structure and S-TRIZ.

Structure	Semantic Relationship	Funding	Article
S + A + O	S= Component A = Function O= Component Component is a physical feature & part of the system	degreeofideality= $\frac{\text{number}\;\text{of}\;\text{functions}}{\text{number}\;\text{of}\;\text{components}}$	A1
S+ (AO)	S = Design Parameters (DPs) AO= Functional Requirements (FRs)	FRs and DPs Serve as Source of Inspiration for Designers & Focus on Customer Needs	A5
S+(AO)	Represent a Function of Technology (S) Forms the Solution. (AO) States the Problem	Function-Oriented Search (FOS)	A10
S + A + O	SAO Function Model: Represent a System (describing the functions of a product/technology) A = Function (directly changes or maintains a controllable or measurable parameter of a (material) object)	Genetic Operation Tree (GOT)	A13
SAO + TRMs	SAO-based PFT maps (Product, Function, Technology). S= Product, Technology AO = purpose function, Effect Function, Partitive Type	(Technology Roadmap) for Strategic Planning and Technology Management	A14
S+(AO)	S = Tool or Method AO = Function SAO Represent (Function Information = Objective + Structure + Effect)	Technology Trends Novel Technologies Potential Infringement	A16
S+(AO)	AO = ‘reasons for jumps’ (RFJ) of TRIZ trends	TRIZ Evolutionary Trends	A17
S+(AO)	(S) Forms the Solution. (AO) States the Problem	Newly Emerging Science & Technology (NEST)	A19
S+(AO)	(S) Forms the Solution. (AO) States the Problem = Function	Technology Opportunity Discovery (TOD) Measured the Semantic Functional Similarities Between Pairs of Products/Technologies	A23
SAO Analysis	Subject = Terms object as O (L, I, T,org)where L is for label, I is for implication, and T is for time and organization. The relationship between objects is described as R(Oi,Oj) Object = materials, techniques, processing methods, products Action = Verb	Problem & Solution (P&S), Problem & Problem (P&P), Solution & Solution (S&S), and Solution & Problem (S&P)	A28
1- Partitive SAO structures 2- Attribute SAO structures	1- Identify Composition of Technologies 2- Identify Properties of Technologies S & O= Component A = Effect or Relationship Between Components S denotes the ‘‘means’’ and A–O denotes the ‘‘end.’’	Identify Technology Opportunities	A32
S+(AO)	(S) Forms the Solution. (AO) States the Problem SAO Represent Functions of Technology and Describe a Relationship Between Components	Identifying R&D Partners	A33
Combines SAO structures with bibliometric analysis	Verbs That Express the Meaning of Requirements = For Example, ‘‘Improve’’, ‘‘Stabilize’’, ‘‘Enhance’’. S, O = components, Problem A = Function, Solution	Requirement-Oriented Core Technological Components' (Technology Process, Operation Method, Function) Identification. Use in Monitoring and Forecasting New Technologies	A34
SAO Network	Subject (node) – Action (edge) – Object (node) calculate the strength of the relationship between nodes, structural holes	Technology Trend Analysis	A38
SAO	(S) = Technical Problem Description, (AO) = Technical Scheme to Solve the Problem	Technology Demand	A41
Subject–Action–Object–others (SAOx)	S = designative terms, AO = TRIZ 39 engineering parameter S and O are set to an invention and engineering parameter, A is used to classify the information from SAOs into two categories according to the relationships between S and O: 1) elements/fields and 2) purposes/effects.	Technology Trends and Explore Technology Opportunities	A42
SAO2Vec	SAO = Determine the Key Technology, Represent the Relationship Between Elements of that Technology, and Present a Variety of Technical Information.	Analysis of Technical Documents	A45
SAO Analysis	S= Solution AO= Problem	Technology Assessment	A54

Although SAO can be applied in various fields of technology and provides fruitful information, further contribution to extracting more solid technical knowledge is necessary. For instance, SAO only focuses on three elements of a sentence, while the rest of the sentence may reveal more details about a system such as purpose, effect, and field, which are often not captured efficiently [71]. SAOs are also unable to identify which components are important in a system [19] or which components belong to the supersystem and main system.

Additionally, “term clumping” method by taking advantage of NLP techniques has been utilized in cleaning and clustering large collections of technical text documents such as patents to obtain information and knowledge in a specific technical domain [61,62,71]. It integrates numerous NLP techniques such as removing stop words and constructing synonym lists, fuzzy set matching, TF-IDF, and PCA [47,52,56,57,60,61].

### 3.5 Data mining and pattern discovery with ML algorithms

**Fig. 10:**
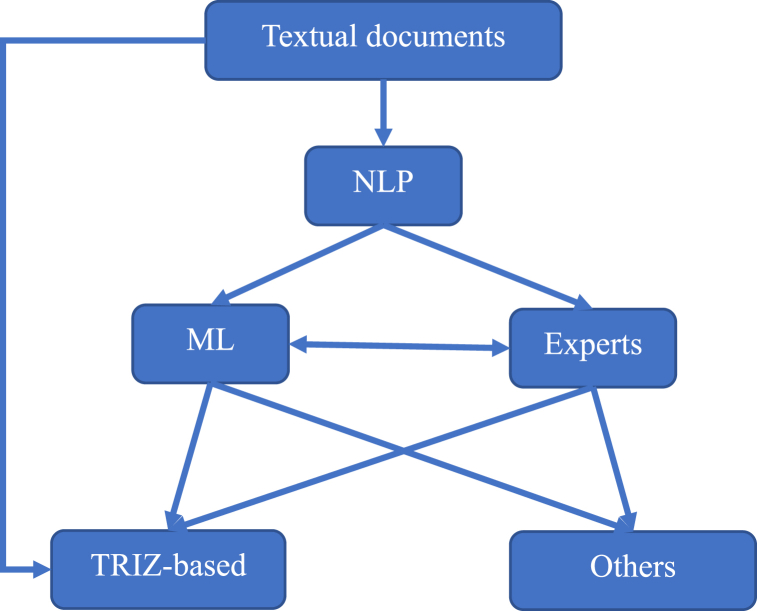
Various type of text analysis in S-TRIZ.

The end goal of S-TRIZ is to automate the manual processes of analyzing, simplifying, and visualizing various TRIZ methods to demonstrate the characteristics of a system in depth. The different types of text analysis procedures in Fig. 10 explain the diversity of studies that were identified during the SLR. Whether information management is based on TRIZ or otherwise is still an area of debate among researchers as it is highly dependent on their subject matter experts. For instance, Verhaegen [104] believed that, notwithstanding TRIZ being categorized as design-by-analogy, novice practitioners face difficulties in interpreting information by analogy.

Therefore, a method for automating the identification process is required. Product aspects for design-by-analogy without considering TRIZ methods have been proposed [104]. However, the aforementioned study introduced a general definition for problem-solving concepts in TRIZ without considering the various tools and fundamental innovative definitions within TRIZ theory.

In fact, there are number of studies that focus on automation of technical document analysis without consideration of TRIZ concepts such as identification of core technologies from patents related to fuel cell vehicle [105], pure research on evaluation of main factors for selecting keywords for patent analysis [106], development of topic modelling framework for ST&I analysis and prediction in context of big data [17], clustering patents over time known as patent lane to identify similarity patterns among patents [107], applied generative topographic mapping method with keyword vectors to identify promising technology opportunities [108], semantic patent analysis applied to detect emerging technologies in the field of camera technology management [109], discovering a type of patent with novel innovation opportunities in the case of Telehealth by using NLP techniques [110], a combination of two approaches namely key-graph based and index-based validation to recognize promising technological innovation [111], clustering and identifying potential opportunities between scientific and technological fields experimented in smart health monitoring [112], quantitative analysis using text mining to detect patent infringement automatically for Nintendo [113], a novel method to quantitatively assess the significance of function score in the area of technology in a determined trend based on genome sequencing [114], R&D project development improvement in China's construction industry through the cross-domain function and its semantical trend analysis [85], and so forth.

The major reasons why the aforementioned papers omitted the usage of TRIZ were the claim that it was rigid, difficult to comprehend, had a limited scope of problem-solving, and demanded expert interventions [85,104,115]. Nevertheless, these claims are debatable as the chosen 57 papers in Table 4 have successfully applied TRIZ fundamentals, and the principles of TRIZ may be modified to suit different engineering system requirements. In addition, TRIZ provides a vivid innovation roadmap and detailed problem-solving methods that can be utilized by both seasoned practitioners and beginners [79,[116], [117], [118]].

In this section, knowledge discovery from text (KDT) [31] algorithms and techniques include data mining and pattern discovery with ML applied in S-TRIZ, covering 57 chosen papers. Table 7 categorizes KDT algorithms and techniques for deep learning, supervised learning, and unsupervised learning.

**Table 7:** ML algorithm applied in S-TRIZ.

ML	Deep learning					Supervised learning				Unsupervised learning		
Algorithms	CNN	ANN	Bi-LSTM	RNN	CRF	SVM	NB	DT	LR	skip-gram	*k*-means	EM
A1	*					*	*					
A2												
A4						*	*	*				
A5		*				*						
A7												
A8												
A11												
A12												
A13												
A14												
A15												
A16											*	
A17												
A19												
A21												
A23												
A24						*	*	*				
A25												
A26												
A27												
A28											*	
A29											*	
A30							*					
A31												
A32												
A33												
A34												
A35												
A38												
A39												
A40												
A41	*		*	*	*	*						
A42												
A43												
A44		*	*		*							
A45										*		
A46												
A47							*					*
A48			*		*						*	
A49												
A50						*	*		*			
A51												
A52												
A53			*									
A55		*										
A56												
A57									*			

**Abbreviation used in this table:** CNN: convolutional neural network, ANN: artificial neural network, Bi-LSTM: bidirectional long short-term memories, RNN: recurrent neural network, CRF: conditional random fields, SVM: support vector machine, NB: naïve bayes, DT: decision tree, LR: logistic regression, EM: expectation and maximization.

Deep learning is a type of supervised ML that leverages neural-network algorithms to train large datasets. The ANNs functions were simulated from a human brain with multiple layers of interconnected neuron webs. Deep learning enriches NLP tasks by creating the patterns to extract and classify the technical features.

Supervised learning algorithms are applied to labelled datasets to classify the words extracted from textual documents. Texts were labelled using tags or annotations for further classification. For instance, we can determine the subject, verb, and adverb over whole sentences using POS tagging to extract SAOs. Subsequently, similar SAOs are classified by training their lexical tags using supervised algorithms. Supervised classification is either a classification that assigns test datasets into predefined categories accurately or a regression that understands the relationship between dependent (response variable) and independent (predictor) variables. The classification of patent documents based on IPC, metadata, and bibliographic information is a challenging area for data scientists [92].

Unsupervised learning is applied to datasets that are not assigned to labels or classes. Clustering algorithms are used for unlabeled texts or documents to group them into similar sets depending on their relevance.

Table 8 categorizes the KDT algorithms for word embedding, collaborative filtering, dimensionality scaling, network modelling, and topic modelling.

**Table 8:**
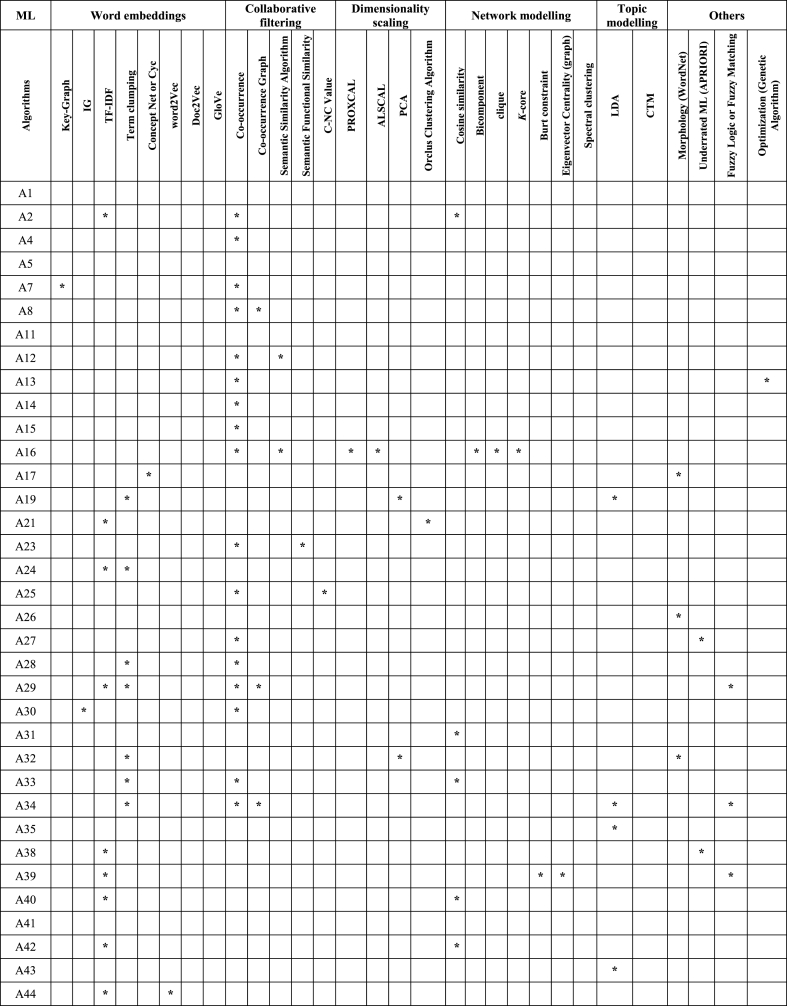

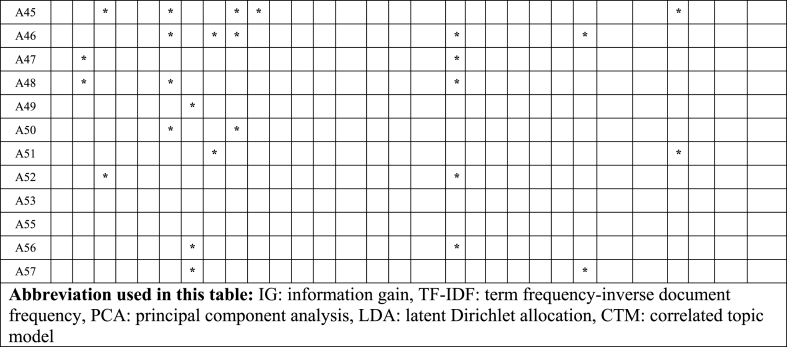
AI techniques applied in S-TRIZ.

Word embedding is the most critical procedure in KDT because of the importance of translating human language into machine language. The outcome of word embedding can be used as an input for the ML algorithms listed above. Collaborative filtering encompasses recommendation techniques, such as co-occurrence, which is applied in most studies.

Co-occurrence verifies the frequency of the two determined words appearing together in textual documents. The distribution of words in documents represents dimensionality. Unnecessary words may lead to noise during the analysis process, particularly in high-dimensional textual datasets. Dimensionality reduction is a common technique used to increase the quality of statistical analysis. The application of the above-mentioned algorithms and techniques faces difficulties in removing dimensionality without a negative impact on the end results. Moreover, multidimensional scaling (MDS) is a statistical model that aims to reduce the complexity of high-dimensional datasets from the aspect of similarity measurements. MDS is beneficial for discovering technology or components similar to the experimental TRIZ tools.

Network modelling or text graphs are visual representations of the synergy or relationship between the extracted keywords. A graph is constructed of nodes, which are terms and edges that represent the relationship between nodes. Visualizing information within textual documents is a trending scope among keywords and SAOs to discover new knowledge. Topic modelling is a well-known unsupervised ML algorithm that tries to discover abstract “topics” by clustering words automatically. In linguistics, morphology refers to the grammatical construction of words and sentences. WordNet is a widely used lexical dictionary.

Exploiting linguistic techniques such as semantic relationships (meronym/holonym) or (hypernym/hyponym) are areas that researchers have used to automatically construct technical morphology. The Apriori algorithm applies prior knowledge to identify the frequency of the determined words in a dataset for the Boolean association rule. However, Apriori is not recommended because it demands high-capacity memory, and its performance is low and inefficient when using large amounts of data.

Fuzzy matching techniques provide further training to identify the similarity between two words, strings, or text entries. For example, fuzzy matching is effective in identifying the extent to which two engineering components or technologies are approximately similar. Evolutionary algorithms, such as genetic algorithms, are applied to the optimization problem based on a heuristic search. A genetic operation tree (GOT) was applied to construct an operation tree (OT) based on SAOs and then translated into a GA genotype as a self-evolutionary model for the automated generation of innovative technology [41]. However, evolutionary algorithms have yet to be developed for comprehensive text analysis, which reflects the requirement for more thorough research.

### 3.6 Interpretation and evaluation of S-TRIZ

**Fig. 11:**
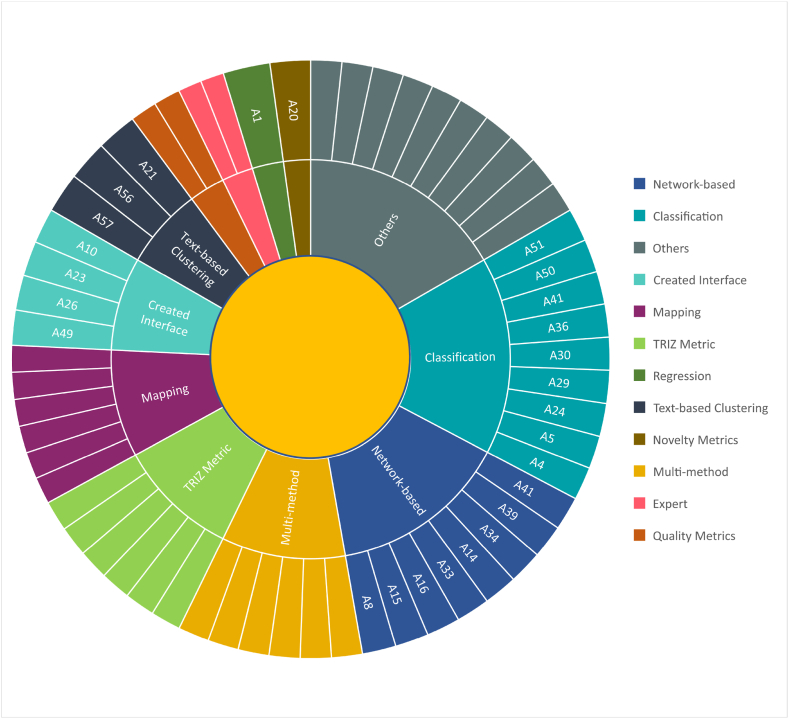
Categories of evaluation performance in S-TRIZ.

In S-TRIZ-based research, performance measurements and indicators to evaluate algorithms are very diverse. The various evaluation performances identified in this paper were classified into ten different categories as illustrated in Fig. 11. TRIZ metrics predefined by Altshuller for assessing technology maturity on an S-curve using indicators such as profitability and cost reduction of products, patents that utilize specific technology, or measuring the degree of novelty [31,32]. For further improvement of products, TRIZ evolution trends adopted valuable criteria to evaluate potential technologies in patents [8,45]. TRIZ metrics are commonly visualized on a radar plot to depict the status of technologies before analyzing further with experts.

Evaluating text classification can be conducted either with schemes that are technology driven [70] such as IPC and united states patent classification (UPC) codes, or TRIZ-based schemes which classify patents based on the Contradictions Matrix and the Inventive Principles [52]. There are two main challenges in evaluating text classification: (1) the lack of protocols and standards for collecting data, and (2) the inability to distinguish various performance measures in multiple experiments [102]. The common indicators for classification assessments are accuracy [58], recall, precision, and F-measure [57] that are measured by using confusion matrix [102]. The accuracy of a regression model can be attained if the target value for to determine features that have low error.

The metrics of accuracy, precision, recall, and F-measure, commonly employed for classification assessment, can be computed as elucidated by Ref. [119]. The four abovementioned indicators in some cases can also be used in clustering assessments in the way Berdyugina and Cavallucci [83] computed statistic measures for contradiction identification versus human extraction. Indexes such as correlation coefficient *R*^*2*^ have been used to measure the level of the invention [30,39]. Several other correlation coefficient measurements including mean squared error (MSE), root mean squared error (RMSE), mean absolute error (MAE) and relative absolute error (RSE) can be used in linear regression [120]. Statistical analysis can be used to assess indicator performance, namely by using *t*-test and correlation analysis [48]. The former aims to make compare the mean value as a way to distinguish various datasets. On the other hand, correlation analysis measures the linkage of predicted and actual values. For instance, a *t*-test can be used to assess the average novelty of design ideation in an experiment on a student's mind [48]. In another experiment, after experts annotated the data, Cohen's kappa coefficient was applied to measure inter-rater reliability as statistic performance [65]. The performance of clustering results for papers and patent corpuses are evaluated by determining cluster sparsity coefficients presented by ORCLUS [49].

Network-based evaluation represents a quantitative relationship among TRIZ technical elements such as functions, technologies, products, etc [69]. The network constitutes of elements such as structural nodes and relationships between elements that connect the links [62]. There are various types of network analysis such as thesaurus network [37], citation network, function–behavior–state network that refers to components of a device [43], and patent networks which are based on semantic similarity amongst them [44], innovation networks which represent the technological similarity between problems and solutions [61], SAO networks which identify relationships between subjects (noun), actions (verb) and objects (noun) to discover technical relationships between them [42,61,62,67]. Nevertheless, the indicators for assessing these various types of networks are (1) centrality (closeness-centrality), (2) density, (3) cohesion index and (4) structural holes. Recently [121], proposed the inverse problem graph (IPG) method in which five types of problems were predefined from the initial analysis of the inventive design. This method was inspired by inventive design method (IDM). IDM framework is a complementary of TRIZ knowledge which applies Pugh's theory or graph theory [87].

Technology Roadmapping (TRM) is a graphical and visual tool that shows industrial information such as materials, products, technologies, components, and so on over time [50,56]. TRM construction can be expert-based, computer-based and hybrid-based [50] which are known as the qualitative, quantitative and hybrid (term/topic-based, P&S pattern-based, fuzzy set-based) method respectively [47,56]. Additionally, TRM was extended by Wang [46] to visualize recursive object model (ROM) and function–behavior–state (FBS) diagram in two-dimensional maps. Patent mapping was? presented by Ref. [70] to select promising topics concerning elements/fields and purposes/effects. A tree model based on TRIZ is another type of mapping that serves to construct concept design [72]. Evaluation of TRM-based methods conducted by experts that define specific indicators regarding to case study development.

A web-based interface is designed to verify the feasibility of a TRIZ tool called function-oriented searching patent by conducting case studies [38]. User interface prototyped by Yoon [51] to assist? system administrators in discovering function-based technology opportunities based on current technological capability. A graphic user interface was developed to indicate the applicability and validity of wordnet-based morphology for ideation [54]. For further R&D evaluation, technology domain experts should examine the validity of interface systems by conducting case studies.

There are some other evaluation techniques which do not belong to any of the above groups and require technical assessments. For instance, for TRIZ-based innovation evaluation, Yu [41] suggested domain experts should evaluate functionality, constructability, and cost effectiveness in the first step and then conduct assessment of real-world application performance. In another experiment, to quantify the potential value of product opportunities, some indicators such as confidence in association rules and the importance of conditional/consequent products presented based on firm's internal capabilities for each product [55]. Novel evaluation indicators are suggested to measure technological feasibility which include (1) magnitude index as a quantitative indicator, (2) importance index as a quantitative indicator, and (3) growth trend index as a qualitative indicator [60]. Kang [63] conducted an actual case study to evaluate market sales data and functional descriptions. In a different study, ISO 9241-11 standard (effectiveness and efficiency) as a quantitative method was used to measure the performance of TRIZ-based inventive problem solving [68]. On the other hand, the feasibility of generating? ideas through morphological matrix on unified structured inventive thinking (simplified TRIZ) to be evaluated further with expert's knowledge [73]. Graph based clustering method known as spectral clustering that applies eigengap heuristic algorithm to calculate the optimal number of groups *k* has been used to evaluate the accuracy of patent clustering based on SAO vectors [19]. Recently, to evaluate candidate terms extracted from patents, unit-hood which implies the degree of strength or stability of syntax combinations, and term-hood which refers to how probable the word that calculated as the *C-value* [74]. Finally, the quantitative outcome-driven innovation (ODI) method is capable of evaluating the importance and satisfaction of technology opportunity [22].

## 4 Results and discussion

Implementing S-TRIZ appropriately has impressive performance in decision-making in R&D projects and industrial development. R&D strategy and management planning include emerging science and technology; forecasting technology; managing innovation; planning product-oriented technology; studying the correlation between science, technology, and innovation; identifying potential opportunities; classifying patents; developing new products; solving problems; and evolving technology.

**Fig. 12:**
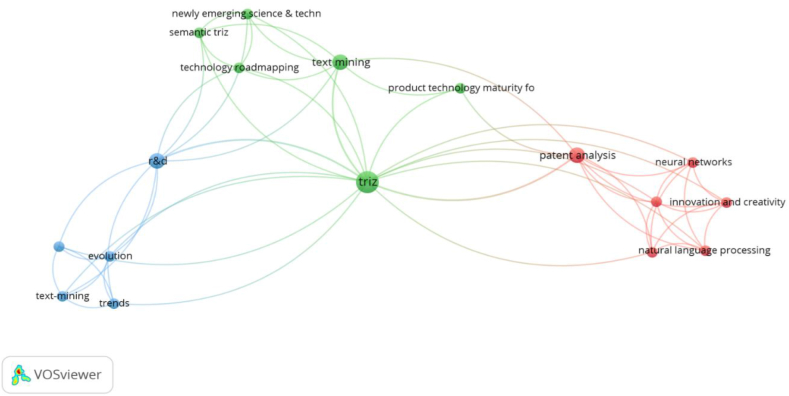
Co-occurrence of selected articles.

In this study, the title, abstract, and keywords of selected articles in Table 4 were analyzed with VOSviewer and are illustrated in Fig. 12, which shows the frequency probability of two terms occurring simultaneously.

Fig. 12 clearly portrays the interconnection of TRIZ with TM and NLP techniques in various R&D studies. Accordingly, the details of the literature review provided in this study will help engineers, designers, domain experts, and innovators on different measures to apply NLP techniques in defining TRIZ concepts semantically and how to improve the process of analysis automatically.

Although some scholars believe that TRIZ concepts are complicated to learn, the results of this study justify the application of ML and NLP, which can eliminate barriers. Various TRIZ tools and continuous enhancements by engineers over the past years have increased their significance in developing engineering systems and furcating technologies. However, there is a lack of research on the development of various TRIZ tools for integrating ML and NLP. This means that the research has limited the general concepts of problem-solving definitions, inventive principles, level of invention, and contradiction matrices that are not adequately developed. For instance, the TRIZ evolutionary trend needs to be improved and developed with respect to the automation process with ML techniques and integrated with various data resources [122]. TRIZ-evolutionary approach has the potential to track development of a system from contradiction to contradiction and provide high-performance solutions by eliminating contradictions [123]. However, this study implies that most research conducted so far strongly relies on manual intervention by experts. This is further supported by Ref. [124] who indicated that TRIZ fundamentally relies on human cognitive mechanisms with less involvement of digital intervention. Furthermore, TRIZ is more skewed towards empirical evidence with a lack of emphasis on scientific theories, and it portrays a lack of comprehensive development to meet the evolving requirements of TRIZ users.

Regarding miscellaneous data resources, the results indicate that patents are the main resources in TRIZ projects. However, there are several limitations, such as studies that combine various datasets within patent fields, or combination of different types of datasets, such as web and social networks. Another limitation is the linguistic diversity of the patent databases.

ML techniques are widely applied in pattern discovery to facilitate research and automate some aspects of studies. Most studies are limited to a specific case study, indicating that they cannot be generalized across other case studies. This was validated by Ref. [81] which illustrated that there is a dire need to increase research on TRIZ and neural networks to accommodate the collection of training data and to create synergy between neural networks and TRIZ. This study presents the techniques and algorithms that have been applied in some areas but are yet to be applied comprehensively. For instance, the classification of patents based on TRIZ concepts requires more experiments in the case of supervised and unsupervised learning. Although these techniques enable scholars to analyze huge amounts of data through big data analysis, there is no solid framework in this area. Additionally, there is still the limitation of full sentence studies, where most of them are restricted to keyword-based or SAO-based studies, which may lead to misinterpretation in certain cases.

Evaluation and interpretation of studies in some efforts were unique and required expert-based knowledge. In some cases, TRIZ domain experts were fundamental, and in other instances, computer science experts were pivotal in reviewing the assessments. Automation of the evaluation process should be considered in future research.

In addition to the scope that has been discussed in this review paper, there are various ongoing related works that researchers have put forward at various conferences. Ni, Samet [125] proposed patent ranking method to achieve inventive solutions from different domains by using LSTM neural networks and XLNet neural networks in the NLP field. In another study, inventive design method matching was introduced in combination with XLNet to construct links between problems and partial solutions [126]. In another study, TRIZ reasoning was reproduced using deep learning techniques to replace the lack of scientific theories in the implementation of TRIZ articulated in Ref. [127]. To prioritize the initial problem in the early phase of inventive design, Hanifi, Chibane [128] applied integration of failure mode effect analysis (FMEA) into the IPG method. Guarino, Samet [86] presented a semi-supervised idea as a patent generative adversarial network to combine multilevel classifiers (sentences and documents) to improve the performance of information extraction from patents. To facilitate the application of the TRIZ contradiction matrix, Berdyugina and Cavallucci [129] utilized the antonym identification technique to automatically extract potential contradictions within a patent. Additionally, a new approach was developed to present a contradiction matrix corresponding to the technical field in real-time by applying NLP techniques within a patent [130]. Berduygina and Cavallucci (2020) discussed an automated method for extracting IDM-related information using NLP was discussed by Ref. [131]. To automate the technical feature extraction of the TRIZ contradiction matrix, Zhai, Li [118] suggested the Doc2Vec model to create the semantic space of patent text. The accuracy of their model was 87%, which reflects an improvement in comparison with the baseline model. Yu [132] adopted hierarchical structured LSTM for TRIZ-Based Chinese patent classification and compared the results with bidirectional encoder representations from transformers (BERT) and other ML algorithms. The results illustrate improvements in “innovation in product design” classification tasks in area under curve score, as opposed to other models.

## 5 Conclusion

In the culmination of our study, a thorough and comprehensive systematic literature review on S-TRIZ analytics has unfolded, highlighting the imperative for in-depth exploration within the realms of TRIZ domains and pivotal concepts, including philosophy, methodology, and tools. This research underscores the critical intersection of insights from both TRIZ experts and the realm of data analytics. With a clarion call for advancement, we advocate for the refinement of existing models and methodologies. This pursuit aims not only to foster practical development, innovation, and production but also to empower engineers seamlessly integrating computer-aided techniques with the rich tapestry of TRIZ principles.

Additionally, we engage in an extensive exploration of the limitations and challenges inherent in S-TRIZ development. While TRIZ serves as a valuable guide for accessing creative solutions, its efficacy is contingent on process automation for user-friendly applications. Notably, 62% of studies centre on existing TRIZ tools, underscoring the necessity to not only refine existing tools but also prioritize the development of essential tools, such as TESE. The diversity of databases, ranging from patent resources like USPTO to academic research and online information, highlights the critical need for their integration and analysis with AI. Although studies indicate a preference for syntactic and keyword-based analyses over sentence-based SAO analyses, advancements in NLP and AI, exemplified by BERT, signal a transformative shift. The selection of ML and AI techniques remains a nuanced challenge, emphasizing the need for careful consideration in specific tasks. Lastly, the most intriguing facet lies in Interpretation and Evaluation, where visualization techniques, including graph-based diagrams, and verification assessments, such as accuracy and precision, are widely applied.

Finally, S-TRIZ, as an integration of computer-aided techniques conforming with TRIZ concepts, demonstrates applicability in conceptualizing innovation across interdisciplinary fields such as auto-remanufacturing, sustainability, recycling, cost-effective production, and robotics.

## Additional information

No additional information is available for this paper.

## CRediT authorship contribution statement

**Mostafa Ghane:** Conceptualization, Data curation, Formal analysis, Investigation, Methodology, Resources, Software, Validation, Visualization, Writing – original draft, Writing – review & editing. **Mei Choo Ang:** Formal analysis, Funding acquisition, Methodology, Project administration, Supervision, Validation. **Denis Cavallucci:** Conceptualization, Methodology, Resources, Supervision, Validation, Visualization. **Rabiah Abdul Kadir:** Funding acquisition, Project administration, Supervision, Validation. **Kok Weng Ng:** Conceptualization, Methodology, Supervision, Validation. **Shahryar Sorooshian:** Funding acquisition, Methodology, Project administration, Resources, Validation, Visualization.

## Declaration of competing interest

The authors declare that they have no known competing financial interests or personal relationships that could have appeared to influence the work reported in this paper.
